# Efficient Approximation of Statistical Significance in Local Trend Analysis of Dependent Time Series

**DOI:** 10.3389/fgene.2022.729011

**Published:** 2022-04-26

**Authors:** Ang Shan, Fang Zhang, Yihui Luan

**Affiliations:** ^1^ Research Center for Mathematics and Interdisciplinary Sciences, Shandong University, Qingdao, China; ^2^ Postdoctoral Programme of Zhongtai Securities Co. Ltd, Jinan, China

**Keywords:** local trend analysis, dependent time series, statistical significance, Markov chain model, spectral decomposition theory

## Abstract

Biological time series data plays an important role in exploring the dynamic changes of biological systems, while the determinate patterns of association between various biological factors can further deepen the understanding of biological system functions and the interactions between them. At present, local trend analysis (LTA) has been commonly conducted in many biological fields, where the biological time series data can be the sequence at either the level of gene expression or OTU abundance, etc., A local trend score can be obtained by taking the similarity degree of the upward, constant or downward trend of time series data as an indicator of the correlation between different biological factors. However, a major limitation facing local trend analysis is that the permutation test conducted to calculate its statistical significance requires a time-consuming process. Therefore, the problem attracting much attention from bioinformatics scientists is to develop a method of evaluating the statistical significance of local trend scores quickly and effectively. In this paper, a new approach is proposed to evaluate the efficient approximation of statistical significance in the local trend analysis of dependent time series, and the effectiveness of the new method is demonstrated through simulation and real data set analysis.

## 1 Introduction

Due to the rapid development of molecular biology technology and the significant reduction to sequencing cost, a large amount of biological time series data has been generated in molecular biological research over the past decade. Among the statistical methods used for time series, local similarity analysis (LSA) has been extensively carried out to identify the correlation between various factors, which can be the genes used in gene expression analysis or operational taxonomic units (OTUs) in metagenomics (Qian et al., 2001; Ruan et al., 2006). Extending the LSA method to the study on the local correlation of repeated time series data, Xia et al. (2011) proposed the extended Local Similarity Analysis method(eLSA), where the confidence interval of LSA was constructed by bootstrap. Due to the ease to use allowed by LSA, it has been widely applied in various fields, for example gene expression profiling (Ji and Tan, 2004; Balasubramaniyan et al., 2005), gene regulatory network construction (Madeira et al. (2010)), symbiotic relationship pattern recognition (Beman et al., 2011; Steele et al.. 2011; Goncalves and Madeira, 2014; Cram et al., 2015) etc. Initially, the permutation test is commonly performed to evaluate the statistical significance of LSA, however, both the approximations of statistical significance and permutation test require the assumption that the time series are independent identically distributed (i.i.d.), which can be violated in most time series data. In order to analyze the statistical significance of LSA for stationary time series, an approach based on moving block bootstrap was proposed by Zhang et al. (2018), and it is referred to as Moving Block Bootstrap LSA (MBBLSA). To assess statistical significance of LSA for stationary time series data, Zhang et al. (2019) developed a theoretical method, which is known as Data Driven LSA (DDLSA). According to DDLSA, long run variance estimated by a nonparametric kernel method is applied to adjust the asymptotic theory of LSA, on the basis of which the limit distribution of LS score for stationary time series can be obtained.

As suggested by Ji and Tan (2004), the degree of similarity shown by rising, unchanged, or falling trends in time series data can be taken as another indicator of the correlation among various biological factors, which is known as local trend analysis (LTA). In LTA, local similarity analysis is performed on the transformed trend sequence, and the corresponding similarity measure is referred to as the local trend score. Local trend analysis is an extension of local similarity analysis, which can better preserve the changing trend of time series. In addition, the discretization of the original sequence can transform some non-stationary time series into stationary Markov series, which is a big advantage of local trend analysis. He and Zeng (2006) applied dynamic programming algorithm to calculate this value, and then conducted permutation test to evaluate statistical significance. Currently, LTA has been widely adopted in many biological fields, including gene association network (He et al.. 2012; Goncalves et al., 2012; Seno et al., 2006; Skreti et al.. 2014) and transcription factor network (Wu et al., 2010). Nevertheless, it takes long to evaluate the statistical significance of local trend analysis through permutation test. In this case, bioinformatics scientists have shifted attention to exploring how the statistical significance of local trend scores can be evaluated quickly and effectively. By extending the statistical significance evaluation method of local similarity analysis theory to local trend analysis, Xia et al. (2015) developed the statistical significance evaluation method of local trend analysis. However, this method is effective only when the original sequence is independent and identically distributed. On the basis of this and prior studies, this paper improves the approximation method proposed by Xia et al. to develop a general method of statistical significance evaluation for local trend analysis.

This paper is organized as follows. In Section 2, an introduction is made of the concept of local trend analysis, and a general method of theoretical evaluation regarding the statistical significance of local trend scores is proposed. In Section 3, the effectiveness of the new method is demonstrated by simulation and real data analysis. Finally, the conclusions and future work are indicated in Section 4.

## 2 Material and Methods

### 2.1 Introduction to Local Trend Analysis

The first step in local trend analysis is to convert time series data into a change trend sequence. In general, if the change trend is indicated by two states, decline and rise, the change trend state set can be set as Σ = (*D*, *U*) or Σ = (−1, 1). If the change trend is indicated by three states decline, unchanged and rise, the change trend state set can be set as Σ = (*D*, *N*, *U*) or Σ = (−1, 0, 1). Undoubtedly, a collection with more changing trend states can be chosen, but it is rare in practice. For a given time series *X*_1_, *X*_2_, …, *X*_*n*_, they can be converted into $d_{i}^{X}\left(i=1,2,\ldots ,n-1\right)$ as follows:

when *X*_*i*_ ≠ 0,

$$d_{i}^{X}=\left\{\begin{array}{cc} 1 & \text{if}\;\frac{X_{i+1}-X_{i}}{\left|X_{i}\right|}\geq t\\ 0 & \text{if}\;-t<\frac{X_{i+1}-X_{i}}{\left|X_{i}\right|}<t\\ -1 & \text{if}\;\frac{X_{i+1}-X_{i}}{\left|X_{i}\right|}\leq -t \end{array}\right.,$$ (1)

where *t* ≥ 0 is a threshold to determine whether there is a trend of change; when *X*_*i*_ = 0,

$$d_{i}^{X}=\left\{\begin{array}{cc} 1 & \text{if}\;X_{i}=0,X_{i+1}>0\\ 0 & \text{if}\;X_{i}=0,X_{i+1}=0\\ -1 & \text{if}\;X_{i}=0,X_{i+1}<0 \end{array}\right..$$ (2)

When *t* = 0, $d_{i}^{X}$ involves only two states, and the change trend state set is Σ = (−1, 1); when *t* ≠ 0, $d_{i}^{X}$ involves three states, and the change trend state set is Σ = (−1, 0, 1). It is assumed that two time series *X*_*t*_ and *Y*_*t*_ are of the same length, *t* = 1, 2, …, *n*. First of all, *X*_*t*_ and *Y*_*t*_ are converted into tred series $d_{i}^{X}$ and $d_{i}^{Y}$ , *i* = 1, 2, …, *n*−1. Given the maximum time delay *D* > 0, the local similarity analysis is conducted on the transformed trend sequence $d_{i}^{X}$ and $d_{i}^{Y}$ to obtain the local trend score *LT*(*D*), i.e.,

$$LT\left(D\right)=\underset{0\leq i,j,k\leq n;|i-j|\leq D}{\max}\left|\overset{k-1}{\underset{l=0}{\sum }}d_{i+l}^{X}d_{j+l}^{Y}\right|.$$ (3)

### 2.2 Statistical Significance Analysis of Local Trend Score

After the local trend score is obtained, it is necessary to evaluate its statistical significance which can be estimated by means of permutation test. In the permutation test, however, only the *p* value obtained by fully permutating the original data is regarded as an accurate estimate. Since the full permutation is a lengthy process, part permutation is usually selected on a random basis. The *p* value obtained at this time is limited to an approximate estimate. Besides, the *p* value obtained may deviate from the actual *p* value if the number of replacements is too small.

In case that the asymptotic distribution result of the local trend score is obtainable, then the *p* value of the local trend score can be obtained through the limit distribution. Probability statisticians have obtained the asymptotic distribution theory of the local similarity scores of Markov chains with a mean value of 0, finite second-order moment, and finite subset in R (Feller, 1951; Daudin et al., 2003; Etienne and Vallois, 2004), as shown in the following theorem.

Theorem 1 Assume that *Z_i_*, *i* = 1, 2, …, n, Markov chains with a mean value of 0, finite second-order moment, and finite subset in R . Assume $E_{\nu }\left(Z_{1}\right)=0$ , $\sigma^{2}=E_{\nu }\left(Z_{1}^{2}\right)+2\sum_{k=1}^{\infty }E_{\nu }\left(Z_{1}Z_{k+1}\right)$ , where ν is the stationary distribution of *Z_i_*. *S_k_* is the random walk process of *Z_i_*:

$$S_{0}=0,S_{k}=\overset{k}{\underset{i=1}{\sum }}Z_{i},1\leq k\leq n.$$

Let

$$H_{n}=\underset{0\leq i\leq j\leq n}{\max}\left(S_{j}-S_{i}\right)=\underset{0\leq i\leq j\leq n}{\max}\left(Z_{i+1}+\cdots +Z_{j}\right).$$

Then $H_{n}/\left(\sigma \sqrt{n}\right)$ is the convergence in probability of W*, where W* = max_0≤*v*≤1_|*W*_v_|, *W*_t_ is a standard Brownian motion.

Xia et al. (2015) used the Theorem 1 to obtain a theoretical evaluation method of statistical significance for local trend analysis. Different from the theoretical evaluation method of statistical significance for local similarity analysis, in local trend analysis, even if the original sequence *X*_*t*_ is independent, the transformed trend sequence $d_{i}^{X}\;\left(i=1,2,\ldots ,n-1\right)$ is not independent, because $d_{i}^{X}$ and $d_{i+1}^{X}$ both depend on *X*_*i*_. In order to facilitate the use of Theorem 1 to calculate the *p* value of the local trend score, the following assumptions are proposed.

Assumption 1
$d_{i}^{X}$ and $d_{i}^{Y}$ are mutually independent first-order Markov chains, and the product of $d_{i}^{X}$ and $d_{i}^{X}$ is also a first-order Markov chain, namely

$$P\left(d_{i}^{X}d_{i}^{Y}|d_{i-1}^{X}d_{i-1}^{Y},\ldots ,d_{1}^{X}d_{1}^{Y}\right)=P\left(d_{i}^{X}d_{i}^{Y}|d_{i-1}^{X}d_{i-1}^{Y}\right).$$ (4)

Under the Assumption 1, $d_{i}^{X}$ and $d_{i}^{Y}$ are irreducible non-periodic Markov chains, so the theoretical method in Feller (1951), Daudin et al. (2003) and Etienne and Vallois (2004) can be directly applied. Xia et al. (2015) suggested a method of theoretically evaluating statistical significance for local trend analysis, with the approximate *p* value of the local trend score *LT*(*D*) obtained as:

$$P\left(LT\left(D\right)\geq s_{D}\right)=P\left(\frac{LT\left(D\right)}{\sigma \sqrt{n}}\geq \frac{s_{D}}{\sigma \sqrt{n}}\right)\approx L_{D}\left(\frac{s_{D}}{\sigma \sqrt{n}}\right),$$ (5)

where *s*_*D*_ represents the local trend score of *X*_*t*_ and *Y*_*t*_, and the definition of the tail probability distribution function $L_{D}\left(x\right)$ is expressed as follows:

$$L_{D}\left(x\right)=1-8^{2D+1}{\left[\overset{\infty }{\underset{k=1}{\sum }}\left\{\frac{1}{x^{2}}+\frac{1}{{\left(2k-1\right)}^{2}\pi^{2}}\right\}\exp\left\{-\frac{{\left(2k-1\right)}^{2}\pi^{2}}{2x^{2}}\right\}\right]}^{2D+1}.$$ (6)

It can be found out that *σ* ^2^ plays a vital role in the *p* value approximation Eq. 5 of the local trend score, which is referred to as the variance of Markov chain. From the formula $\sigma^{2}=E_{\nu }\left(Z_{1}^{2}\right)+2\sum_{k=1}^{\infty }E_{\nu }\left(Z_{1}Z_{k+1}\right)$ , it can be seen that when the stationary distribution of Markov chain *ν* and *k* step transition probability matrix are known, $E_{\nu }\left(Z_{1}Z_{k}\right)\;\left(k\geq 1\right)$ can be obtained. Thus, *σ* ^2^ can be obtained easily through calculation. Xia et al. presented the display expression of *σ* ^2^ when the original sequence is independent and identically distributed. In practice, however, the original sequence contradicts the assumption of independent and identical distribution. Zhang et al. (2019) proposed an asymptotic statistical significance for local similarity analysis, with the approximate *p* value of the local similarity score *LS*(*D*) similar to *LT*(*D*):

$$P\left(LS\left(D\right)\geq s_{D}\right)=P\left(\frac{LS\left(D\right)}{\omega \sqrt{n}}\geq \frac{s_{D}}{\omega \sqrt{n}}\right)\approx L_{D}\left(\frac{s_{D}}{\omega \sqrt{n}}\right),$$ (7)

where $\omega =\lim_{n\to \infty }\sqrt{\mathrm{var}\left(\sum_{i=1}^{n}Z_{i}\right)/n}$ is referred to as the long-run variance, and $L_{D}\left(x\right)$ is expressed as Eq. 6. Because Markov chains can be regarded as time series, they also satisfy Eq. 7. It is obvious that *ω* for Markov chains is *σ*. Therefore, we can get the statistical significance for local trend analysis of non-independent identically distributed time series if the *σ* ^2^ is obtained.

Next, the formula of *σ* ^2^ is proposed for the local trend score of the time series in general using the spectral decomposition theory of the matrix.

#### 2.2.1 Spectral Decomposition Theorem of Matrix

First, the definition and properties of simple matrix are given.

Definition 1 Let matrix ***A*** ∈ ***C*** *^n×n^*, λ_i_ be the differential eigenvalues of ***A***, *i* = 1, 2, …, s, and the characteristic polynomial of **A** is

$$\det\left(\lambda I-A\right)={\left(\lambda -\lambda_{1}\right)}^{m_{1}}{\left(\lambda -\lambda_{2}\right)}^{m_{2}}\ldots ,{\left(\lambda -\lambda_{s}\right)}^{m_{s}},$$

where $\sum_{i=1}^{s}m_{i}=n$ . Call m_i_ the algebraic multiplicity of the eigenvalues *λ_i_* of the matrix ***A***.

Definition 2 The solution space $V_{\lambda_{i}}$ of the homogeneous equation set ***A**x* = *λ_i_* x (i = 1, 2, …, s) is called the eigenspace of ***A*** corresponding to the eigenvalue *λ_i_*, and the dimension of $V_{\lambda_{i}}$ is called the geometric multiplicity of the eigenvalue λ_i_ of the matrix ***A***.

Definition 3 If the algebraic multiplicity of each eigenvalue of the matrix ***A*** is equal to its geometric multiplicity, then ***A*** is called a simple matrix.

Theorem 2 (Spectral decomposition theorem) Let matrix ***A*** *∈ **C** ^n×n^, λ_i_* be the differential eigenvalues of ***A***, m_i_ is the algebraic multiplicity of λ_i_, i = 1, 2, …, s, then the sufficient and necessary condition of ***A*** being a simple matrix is that there is a unique ***E**_i_ ∈ **C** ^n×n^*, i = 1, 2, …, s, so

1) $\sum_{i=1}^{s}E_{i}=I$ *.*
2) $E_{i}E_{j}=\left\{\begin{array}{cc} E_{i},\; & i=j\\ 0,\; & i\neq j \end{array}\right..$

3) $A=\sum_{i=1}^{s}\lambda_{i}E_{i}$ *.*

#### 2.2.2 Two-State Markov Chain Model

Firstly, the two-state Markov chain model is studied. When *t* = 0, $d_{i}^{X}$ and $d_{i}^{Y}$ , *i* = 1, 2, …, *n*−1 can be obtained by discretizing the original sequence *X*_*t*_ and *Y*_*t*_. Assume that the distribution of the original sequence is symmetrical, and the mean is 0. Also assume that $d_{i}^{X}$ is a first-order stationary Markov chain. Since the original sequence distribution is symmetrical, the stationary distribution of $d_{i}^{X}$ is $P\left(d_{i}^{X}=1\right)=P\left(d_{i}^{X}=-1\right)=1/2$ , $E\left({\left(d_{1}^{X}\right)}^{2}\right)=1^{2}\times \frac{1}{2}+{\left(-1\right)}^{2}\times \frac{1}{2}=1$ . It is assumed that the transition probability matrices of $d_{i}^{X}$ and $d_{i}^{Y}$ are ***T***_*X*_ and ***T***_*Y*_ respectively, as expressed below.


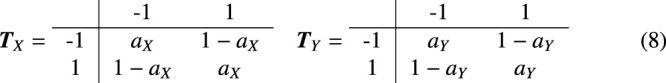


It can be obtained by calculation, $E(d_{1}^{X}d_{k+1}^{X})={\left(2a_{X}-1\right)}^{k}$ , $E\left({\left(d_{1}^{X}\right)}^{2}\right)=E\left({\left(d_{1}^{Y}\right)}^{2}\right)=1$ , $E\left(d_{1}^{Y}d_{k+1}^{Y}\right)={\left(2a_{Y}-1\right)}^{k}$ (Supplementary Material S1). Under the null hypothesis that *X*_*i*_ and *Y*_*i*_ are uncorrelated,

$$\begin{array}{cc} \sigma^{2} & =E\left({\left(d_{1}^{X}d_{1}^{Y}\right)}^{2}\right)+2\overset{\infty }{\underset{k=1}{\sum }}E\left(\left(d_{1}^{X}d_{k+1}^{X}\right)\left(d_{1}^{Y}d_{k+1}^{Y}\right)\right)\\ & =E\left({\left(d_{1}^{X}\right)}^{2}\right)E\left({\left(d_{1}^{Y}\right)}^{2}\right)+2\overset{\infty }{\underset{k=1}{\sum }}E\left(d_{1}^{X}d_{k+1}^{X}\right)E\left(d_{1}^{Y}d_{k+1}^{Y}\right)\\ & =1+2\overset{\infty }{\underset{k=1}{\sum }}{\left(2a_{X}-1\right)}^{k}{\left(2a_{Y}-1\right)}^{k}\\ & =1+2\times \underset{k\to \infty }{\lim}\frac{\left(2a_{X}-1\right)\left(2a_{Y}-1\right)-{\left(2a_{X}-1\right)}^{k+1}{\left(2a_{Y}-1\right)}^{k+1}}{1-\left(2a_{X}-1\right)\left(2a_{Y}-1\right)}\\ & =1+2\times \frac{\left(2a_{X}-1\right)\left(2a_{Y}-1\right)}{1-\left(2a_{X}-1\right)\left(2a_{Y}-1\right)}\\ & =\frac{1+\left(2a_{X}-1\right)\left(2a_{Y}-1\right)}{1-\left(2a_{X}-1\right)\left(2a_{Y}-1\right)}. \end{array}$$ (9)

thus, when *t* = 0, the *p* value of the local trend score *LT*(*D*) is written as

$$P\left(LT\left(D\right)\geq s_{D}\right)=L_{D}\left(\frac{s_{D}}{\sigma \sqrt{n}}\right),$$ (10)

where *s*_*D*_ indicates the local trend score of *X*_*i*_ and *Y*_*i*_, *σ* is obtained using the Eq. 9, and $L_{D}\left(x\right)$ is defined as Eq. 6.

#### 2.2.3 Three-State Markov Chain Model

Secondly, the three-state Markov chain model is studied. When *t* ≠ 0, $d_{i}^{X}$ and $d_{i}^{Y}$ are three-state Markov chains. Similarly, it is assumed that the transition probability matrices of $d_{i}^{X}$ and $d_{i}^{Y}$ are ***T***_*X*_ and ***T***_*Y*_ respectively, as expressed below.


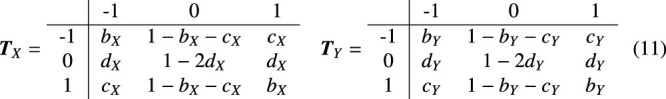


It can be obtained by calculation, $E\left(d_{1}^{X}d_{k+1}^{X}\right)=\varphi_{1}^{X}T_{1,1}^{X,k}+\varphi_{-1}^{X}T_{-1,-1}^{X,k}-\varphi_{1}^{X}T_{1,-1}^{X,k}-\varphi_{-1}^{X}T_{-1,1}^{X,k}$ , $E\left({\left(d_{1}^{X}\right)}^{2}\right)=\varphi_{-1}^{X}+\varphi_{1}^{X}$ , $E\left({\left(d_{1}^{Y}\right)}^{2}\right)=\varphi_{-1}^{Y}+\varphi_{1}^{Y}$ , $E\left(d_{1}^{Y}d_{k+1}^{Y}\right)=\varphi_{1}^{Y}T_{1,1}^{Y,k}+\varphi_{-1}^{Y}T_{-1,-1}^{Y,k}-\varphi_{1}^{Y}T_{1,-1}^{Y,k}-\varphi_{-1}^{Y}T_{-1,1}^{Y,k}$ (Supplementary Material S2). Under the null hypothesis that *X*_*i*_ and *Y*_*i*_ are uncorrelated,

$$\begin{array}{ll} \sigma^{2} & =E\left({\left(d_{1}^{X}d_{1}^{Y}\right)}^{2}\right)+2\overset{\infty }{\underset{k=1}{\sum }}E\left(\left(d_{1}^{X}d_{k+1}^{X}\right)\left(d_{1}^{Y}d_{k+1}^{Y}\right)\right)\\ & =E\left({\left(d_{1}^{X}\right)}^{2}\right)E\left({\left(d_{1}^{Y}\right)}^{2}\right)+2\overset{\infty }{\underset{k=1}{\sum }}E\left(d_{1}^{X}d_{k+1}^{X}\right)E\left(d_{1}^{Y}d_{k+1}^{Y}\right)\\ & =\left(\varphi_{-1}^{X}+\varphi_{1}^{X}\right)\left(\varphi_{-1}^{Y}+\varphi_{1}^{Y}\right)+2\overset{\infty }{\underset{k=1}{\sum }}\left(\varphi_{1}^{X}T_{1,1}^{X,k}+\varphi_{-1}^{X}T_{-1,-1}^{X,k}-\varphi_{1}^{X}T_{1,-1}^{X,k}-\varphi_{-1}^{X}T_{-1,1}^{X,k}\right)\\ & \left(\varphi_{1}^{Y}T_{1,1}^{Y,k}+\varphi_{-1}^{Y}T_{-1,-1}^{Y,k}-\varphi_{1}^{Y}T_{1,-1}^{Y,k}-\varphi_{-1}^{Y}T_{-1,1}^{Y,k}\right)\\ & =4\varphi_{1}^{X}\varphi_{1}^{Y}+2\varphi_{1}^{X}\varphi_{1}^{Y}\overset{\infty }{\underset{k=1}{\sum }}\left(T_{1,1}^{X,k}+T_{-1,-1}^{X,k}-T_{1,-1}^{X,k}-T_{-1,1}^{X,k}\right)\\ & \left(T_{1,1}^{Y,k}+T_{-1,-1}^{Y,k}-T_{1,-1}^{Y,k}-T_{-1,1}^{Y,k}\right)\\ & =4\varphi_{1}^{X}\varphi_{1}^{Y}+2\varphi_{1}^{X}\varphi_{1}^{Y}\overset{\infty }{\underset{k=1}{\sum }}2{\left(b_{X}-c_{X}\right)}^{k}\times 2{\left(b_{Y}-c_{Y}\right)}^{k}\\ & =4\varphi_{1}^{X}\varphi_{1}^{Y}\left(1+2\underset{k\to \infty }{\lim}\frac{\left(b_{X}-c_{X}\right)\left(b_{Y}-c_{Y}\right)-{\left(b_{X}-c_{X}\right)}^{k+1}{\left(b_{Y}-c_{Y}\right)}^{k+1}}{1-\left(b_{X}-c_{X}\right)\left(b_{Y}-c_{Y}\right)}\right)\\ & =4\left(\frac{d_{X}}{1-b_{X}-c_{X}+2d_{X}}\right)\left(\frac{d_{Y}}{1-b_{Y}-c_{Y}+2d_{Y}}\right)\left(\frac{1+\left(b_{X}-c_{X}\right)\left(b_{Y}-c_{Y}\right)}{1-\left(b_{X}-c_{X}\right)\left(b_{Y}-c_{Y}\right)}\right). \end{array}$$ (12)

Thus, when *t* ≠ 0, the *p* value of the local trend score *LT*(*D*) is expressed as

$$P\left(LT\left(D\right)\geq s_{D}\right)=L_{D}\left(\frac{s_{D}}{\sigma \sqrt{n}}\right),$$ (13)

where *s*_*D*_ represents the local trend score of *X*_*i*_ and *Y*_*i*_, *σ* is obtained using the Eq. 12, and $L_{D}\left(x\right)$ is defined as Eq. 6.

#### 2.2.4 Mixed-State Markov Chain Model

Thirdly, the mixed-state Markov chain model is studied. When *t* ≠ 0, $d_{i}^{X}$ or $d_{i}^{Y}$ is potentially a two-state Markov chain as well. At this time, if $d_{i}^{X}$ and $d_{i}^{Y}$ are both two-state Markov chains, *σ* ^2^ can be estimated using the two-state Markov chain model. The circumstance where only $d_{i}^{X}$ or $d_{i}^{Y}$ is a two-state Markov chain is defined as a mixed-state Markov chain model. Without any compromise on generality, it is supposed that $d_{i}^{X}$ is a two-state Markov chain while $d_{i}^{Y}$ is a three-state Markov chain.

It can obtained by the previous derivation that

$$\begin{array}{cc} & E\left({\left(d_{1}^{X}\right)}^{2}\right)=1,\\ & E\left(d_{1}^{X}d_{k+1}^{X}\right)={\left(2a_{X}-1\right)}^{k},\\ & E\left({\left(d_{1}^{Y}\right)}^{2}\right)=\varphi_{-1}^{Y}+\varphi_{1}^{Y}=\frac{2d_{Y}}{1-b_{Y}-c_{Y}+2d_{Y}},\\ & E\left(d_{1}^{Y}d_{k+1}^{Y}\right)=\varphi_{1}^{Y}T_{1,1}^{Y,k}+\varphi_{-1}^{Y}T_{-1,-1}^{Y,k}-\varphi_{1}^{Y}T_{1,-1}^{Y,k}-\varphi_{-1}^{Y}T_{-1,1}^{Y,k},\\ & \;\;\;=2\left(\frac{d_{Y}}{1-b_{Y}-c_{Y}+2d_{Y}}\right){\left(b_{Y}-c_{Y}\right)}^{k}. \end{array}$$

So,

$$\begin{array}{cc} \sigma^{2} & =E\left({\left(d_{1}^{X}d_{1}^{Y}\right)}^{2}\right)+2\overset{\infty }{\underset{k=1}{\sum }}E\left(\left(d_{1}^{X}d_{k+1}^{X}\right)\left(d_{1}^{Y}d_{k+1}^{Y}\right)\right)\\ & =E\left({\left(d_{1}^{X}\right)}^{2}\right)E\left({\left(d_{1}^{Y}\right)}^{2}\right)+2\overset{\infty }{\underset{k=1}{\sum }}E\left(d_{1}^{X}d_{k+1}^{X}\right)E\left(d_{1}^{Y}d_{k+1}^{Y}\right)\\ & =\frac{2d_{Y}}{1-b_{Y}-c_{Y}+2d_{Y}}+\frac{4d_{Y}}{1-b_{Y}-c_{Y}+2d_{Y}}\overset{\infty }{\underset{k=1}{\sum }}{\left(2a_{X}-1\right)}^{k}{\left(b_{Y}-c_{Y}\right)}^{k}\\ & =\left(\frac{2d_{Y}}{1-b_{Y}-c_{Y}+2d_{Y}}\right)\times \\ & \;\left(1+2\underset{k\to \infty }{\lim}\frac{\left(2a_{X}-1\right)\left(b_{Y}-c_{Y}\right)-{\left(2a_{X}-1\right)}^{k+1}{\left(b_{Y}-c_{Y}\right)}^{k+1}}{1-\left(2a_{X}-1\right)\left(b_{Y}-c_{Y}\right)}\right)\\ & =\left(\frac{2d_{Y}}{1-b_{Y}-c_{Y}+2d_{Y}}\right)\left(\frac{1+\left(2a_{X}-1\right)\left(b_{Y}-c_{Y}\right)}{1-\left(2a_{X}-1\right)\left(b_{Y}-c_{Y}\right)}\right). \end{array}$$ (14)

Thus, when *t* ≠ 0 and the circumstance arises that $d_{i}^{X}$ and $d_{i}^{Y}$ are not both three-state Markov chains, the *p* value of the local trend score *LT*(*D*) is expressed as

$$P\left(LT\left(D\right)\geq s_{D}\right)=L_{D}\left(\frac{s_{D}}{\sigma \sqrt{n}}\right),$$ (15)

where *s*_*D*_ represents the local trend score of *X*_*i*_ and *Y*_*i*_, *σ* is obtained using the Eq. 14, and $L_{D}\left(x\right)$ is defined as Eq. 6.

In summary, the *p* value approximation formula has been obtained for the local trend score of a two-state, three-state or mixed-state Markov chain. Despite a lack of rigorous mathematical proof for the aforementioned *p* value approximation method, it is still discovered that the *p* value obtained using this algorithm is approximately equal to the given significance level by simulation, especially when the sample size is large. Therefore, the results obtained using this method are deemed approximately valid.

#### 2.2.5 Estimation of Markov Chain Transition Probability Matrix

In order to calculate the *p* value of the local trend score, it is essential to estimate the variance *σ* ^2^, and the estimation of the variance depends only on the transition probability matrix of the Markov chain. With the original sequence considered as independent and identically distributed, Xia et al. (2015) deduced the value of parameter in transition probability matrix of the two-state (*t* = 0) and three-state (*t* = 0.5) Markov chain. When the original series are non-independent and identically distributed, however, the estimate is inaccurate. It is detailed below how to estimate the transition probability matrix of a two-state or three-state Markov chain under normal circumstances.

For a two-state Markov chain, since both *T*_−1,−1_ and *T*_1,1_ are equal to *a*, the mean of *n*_−1,−1_/*n*_−1,⋅_ and *n*_1,1_/*n*_1,⋅_ is taken as the final estimate of *a*, that is, $\hat{a}=\frac{1}{2}\left(\frac{n_{-1,-1}}{n_{-1,\cdot }}+\frac{n_{1,1}}{n_{1,\cdot }}\right)$ , where *n*_−1,⋅_ = *n*_−1,−1_ + *n*_−1,1_, *n*_1,⋅_ = *n*_1,−1_ + *n*_1,1_, *n*_*u*,*v*_ represents the number of (*d*_*i*_, *d*_*i*+1_) = (*u*, *v*), *u*, *v* ∈ (−1, 1), *i* = 1, 2, …, *n* − 2.

Likewise, for a three-state Markov chain, since both *T*_−1,−1_ and *T*_1,1_ are equal to *b*, the mean of *n*_−1,−1_/*n*_−1,⋅_ and *n*_1,1_/*n*_1,⋅_ is treated as the final estimate of *b*, that is, $\hat{b}=\frac{1}{2}\left(\frac{n_{-1,-1}}{n_{-1,\cdot }}+\frac{n_{1,1}}{n_{1,\cdot }}\right)$ , where *n*_−1,⋅_ = *n*_−1,−1_ + *n*_−1,0_ + *n*_−1,1_, *n*_1,⋅_ = *n*_1,−1_ + *n*_1,0_ + *n*_1,1_, and *n*_*u*,*v*_ represents the number of (*d*_*i*_, *d*_*i*+1_) = (*u*, *v*), *u*, *v* ∈ (−1, 0, 1), *i* = 1, 2, …, *n*−2. Similarly, the estimate of *c* is $\hat{c}=\frac{1}{2}\left(\frac{n_{-1,1}}{n_{-1,\cdot }}+\frac{n_{1,-1}}{n_{1,\cdot }}\right)$ , and the estimate of *d* is $\hat{d}=\frac{1}{2}\left(\frac{n_{0,-1}+n_{0,1}}{n_{0,\cdot }}\right)$ , where *n*_0,⋅_ = *n*_0,−1_ + *n*_0,0_ + *n*_0,1_.

In this article, the method put forward by Xia et al. is denoted as TLTA (Theoretical Local Trend Analysis), while the method proposed in this paper is referred to as STLTA (Stationary Theoretical Local Trend Analysis).

## 3 Results and Discussion

### 3.1 Simulation

The effects on the correlation test of time series data are explored by conducting Permutation test, TLTA and STLTA respectively. The following three models are commonly used and familiar to researchers, which can better reflect the correlation between two time series, especially the correlation of two time series can be adjusted by changing the coefficient values. In order to study the difference in type I error rate and significance level among different methods under the original hypothesis, simulation data is generated using the following three models: The effects on the correlation test of time series data are explored by conducting Permutation test, TLTA and STLTA respectively. In order to study the difference in type I error rate and significance level among different methods under the original hypothesis, simulation data is generated using the following three models:

1) AR(1) model:

$$\begin{array}{cc} & X_{t}=\rho_{1}X_{t-1}+\varepsilon_{t}^{X},\\ & Y_{t}=\rho_{2}Y_{t-1}+\varepsilon_{t}^{Y}. \end{array}$$ (16)

2) ARMA(1,1) model:

$$\begin{array}{cc} & X_{t}=\rho_{1}X_{t-1}+\varepsilon_{t}^{X}+0.5\varepsilon_{t-1}^{X},\\ & Y_{t}=\rho_{2}Y_{t-1}+\varepsilon_{t}^{Y}+0.5\varepsilon_{t-1}^{Y}. \end{array}$$ (17)

3) ARMA(1,1)-TAR(1) model:

$$\begin{array}{cc} & X_{t}=\rho_{1}X_{t-1}+\varepsilon_{t}^{X}+0.5\varepsilon_{t-1}^{X},\\ & Y_{t}=\left\{\begin{array}{c} \rho_{2}Y_{t-1}+\varepsilon_{t}^{Y},Y_{t-1}\leq -1\;\\ 0.5Y_{t-1}+\varepsilon_{t}^{Y},Y_{t-1}>-1.\; \end{array}\right. \end{array}$$ (18)

Where 0 < |*ρ*_1_|, |*ρ*_2_| < 1, $\varepsilon_{t}^{X}$ and $\varepsilon_{t}^{Y}$ are independent standard normal random variables. All the three models are stationary. For each model, it starts by generating *X*_1_ and *Y*_1_ through the standard normal distribution, before the generation of *X*_*t*_ and *Y*_*t*_, *i* = 2, …, 100, + , *n* using the above-mentioned model. Finally, the first 100 samples are discarded, and the remaining *n* samples are treated as real *X*_*t*_ and *Y*_*t*_. This data generation process is effective in ensuring the stationarity of the time series.

With consideration given to the impact of autoregressive coefficients *ρ*_1_, *ρ*_2_ and sample size *n* on the type I error rate for the different methods with the three models, we choose six different combinations of autoregressive coefficients *ρ*_1_, *ρ*_2_, and respectively take the values of −0.5, −0.5; 0, 0; 0.3, 0.3; 0.3, 0.5; 0.5, 0.5; 0.5, 0.8. For each combination of autoregressive coefficients, the sample size *n* is set to 20, 40, 60, 80, 100, 200. For simplicity, we select the time delay *D* = 0. In all simulations, the significance level is set to 0.05.

When *t* = 0, the original sequence is converted into a two-state Markov chain, and the type I error rates in the AR(1) model of different methods are presented in Table 1. The results show that when *ρ*_1_ = −0.5, *ρ*_2_ = −0.5, neither Permutation test nor TLTA can control the type I error rate even if the sample size *n* is small, and their type I error rates are getting bigger as the sample size increases. At this time, the type I error rate of STLTA gradually approaches the significance level 0.05 with the increase of sample size. When *ρ*_1_ = 0, *ρ*_2_ = 0, *X*_*t*_ and *Y*_*t*_ are all independent and identically distributed sequences, the type I error rates of the three methods are very close to the given significance level, and are getting closer as the sample size increases. When *ρ*_1_ > 0, *ρ*_2_ > 0, the type I error rate of Permutation test decreases with the increase of sample size *n*, and gradually deviates from the significance level 0.05, while the type I error rate of STLTA is closer to the significance level than that of TLTA. For different autocorrelation coefficients, the type I error rates of Permutation test and TLTA show a declining trend with the increase of *ρ*, and they are increasingly deviant from the significance level. By contrast, STLTA shows an upward trend with the rise of *ρ*, and it gradually approaches the significance level, suggesting that STLTA is more suitable for stationary time series data. The performances of these three methods in ARMA(1,1) and ARMA(1,1)-TAR(1) models are shown in the Tables 2, 3 respectively, which are similar to that in the AR(1) model. Under these two models, when *ρ*_1_ = −0.5, *ρ*_2_ = −0.5, *X*_*t*_ is an independent and identically distributed sequence, so the type I error rates of Permutation test, TLTA and STLTA are close to the significance level. In other cases, the type I error rate of STLTA is closer to the significance level than that of TLTA, while the type I error rate of Permutation test gradually gets away from the significance level as the sample size increases.

**TABLE 1:** Type I error rate for different methods (the third to fifth columns) in the AR(1) model when*t* = 0. The first and second columns represent different combinations of autoregressive coefficients and sample sizes. The number of permutation tests is 1,000, the number of repeated simulations is 10,000, and the significance level is *α* = 0.05.

*ρ*_1_, *ρ*_2_	*n*	Permutation test	TLTA	STLTA
−0.5, −0.5	20	0.1413	0.0470	0.0040
	40	0.1444	0.0764	0.0128
	60	0.1378	0.0880	0.0169
	80	0.1472	0.1040	0.0213
	100	0.1380	0.1046	0.0238
	200	0.1465	0.1059	0.0283
0, 0	20	0.0610	0.0170	0.0119
	40	0.0613	0.0270	0.0209
	60	0.0605	0.0311	0.0257
	80	0.0545	0.0363	0.0282
	100	0.0551	0.0360	0.0300
	200	0.0581	0.0367	0.0357
0.3, 0.3	20	0.0518	0.0109	0.0136
	40	0.0451	0.0177	0.0272
	60	0.0475	0.0179	0.0285
	80	0.0408	0.0238	0.0310
	100	0.0435	0.0260	0.0349
	200	0.0428	0.0254	0.0371
0.3, 0.5	20	0.0459	0.0092	0.0135
	40	0.0397	0.0165	0.0288
	60	0.0379	0.0181	0.0314
	80	0.0407	0.0233	0.0334
	100	0.0359	0.0237	0.0354
	200	0.0345	0.0221	0.0424
0.5, 0.5	20	0.0398	0.0091	0.0159
	40	0.0414	0.0159	0.0284
	60	0.0365	0.0176	0.0314
	80	0.0369	0.0199	0.0343
	100	0.0355	0.0213	0.0374
	200	0.0344	0.0215	0.0428
0.5, 0.8	20	0.0412	0.0088	0.0161
	40	0.0388	0.0134	0.0277
	60	0.0338	0.0145	0.0342
	80	0.0319	0.0165	0.0357
	100	0.0337	0.0214	0.0411
	200	0.0314	0.0170	0.0402

**TABLE 2:** Type I error rate for different methods (the third to fifth columns) in the ARMA(1,1) model when*t* = 0. The first and second columns represent different combinations of autoregressive coefficients and sample sizes. The number of permutation tests is 1,000, the number of repeated simulations is 10,000, and the significance level is *α* = 0.05.

*ρ*_1_, *ρ*_2_	*n*	Permutation test	TLTA	STLTA
−0.5, −0.5	20	0.0617	0.0166	0.0112
	40	0.0609	0.0262	0.0219
	60	0.0557	0.0323	0.0289
	80	0.0562	0.0333	0.0267
	100	0.0538	0.0354	0.0311
	200	0.0572	0.0338	0.0329
0, 0	20	0.0444	0.0109	0.0210
	40	0.0463	0.0170	0.0380
	60	0.0455	0.0213	0.0404
	80	0.0422	0.0270	0.0464
	100	0.0397	0.0242	0.0444
	200	0.0428	0.0260	0.0539
0.3, 0.3	20	0.0472	0.0109	0.0240
	40	0.0497	0.0168	0.0426
	60	0.0413	0.0187	0.0404
	80	0.0395	0.0222	0.0421
	100	0.0421	0.0261	0.0545
	200	0.0418	0.0250	0.0559
0.3, 0.5	20	0.0483	0.0095	0.0218
	40	0.0447	0.0172	0.0410
	60	0.0438	0.0198	0.0427
	80	0.0453	0.0230	0.0432
	100	0.0399	0.0240	0.0515
	200	0.0420	0.0231	0.0505
0.5, 0.5	20	0.0503	0.0097	0.0220
	40	0.0409	0.0186	0.0403
	60	0.0455	0.0191	0.0417
	80	0.0445	0.0235	0.0460
	100	0.0399	0.0271	0.0509
	200	0.0342	0.0257	0.0591
0.5, 0.8	20	0.0492	0.0093	0.0202
	40	0.0430	0.0158	0.0337
	60	0.0399	0.0193	0.0372
	80	0.0435	0.0206	0.0366
	100	0.0359	0.0204	0.0418
	200	0.0381	0.0199	0.0462

**TABLE 3:** Type I error rate for different methods (the third to fifth columns) in the ARMA(1,1)-TAR(1) model when*t* = 0. The first and second columns represent different combinations of autoregressive coefficients and sample sizes. The number of permutation tests is 1,000, the number of repeated simulations is 10,000, and the significance level is *α* = 0.05.

*ρ*_1_, *ρ*_2_	*n*	Permutation test	TLTA	STLTA
−0.5, −0.5	20	0.0563	0.0127	0.0119
	40	0.0527	0.0194	0.0220
	60	0.0463	0.0247	0.0282
	80	0.0481	0.0279	0.0285
	100	0.0481	0.0264	0.0291
	200	0.0437	0.0277	0.0341
0, 0	20	0.0437	0.0083	0.0147
	40	0.0436	0.0150	0.0270
	60	0.0393	0.0177	0.0350
	80	0.0412	0.0212	0.0377
	100	0.0354	0.0210	0.0382
	200	0.0362	0.0221	0.0435
0.3, 0.3	20	0.0395	0.0076	0.0172
	40	0.0382	0.0126	0.0332
	60	0.0393	0.0136	0.0349
	80	0.0363	0.0183	0.0385
	100	0.0353	0.0195	0.0411
	200	0.0296	0.0186	0.0470
0.3, 0.5	20	0.0372	0.0068	0.0199
	40	0.0345	0.0128	0.0328
	60	0.0356	0.0137	0.0336
	80	0.0328	0.0174	0.0382
	100	0.0315	0.0208	0.0437
	200	0.0354	0.0184	0.0448
0.5, 0.5	20	0.0343	0.0067	0.0170
	40	0.0338	0.0130	0.0337
	60	0.0305	0.0130	0.0367
	80	0.0319	0.0196	0.0400
	100	0.0309	0.0160	0.0399
	200	0.0251	0.0163	0.0463
0.5, 0.8	20	0.0410	0.0061	0.0176
	40	0.0316	0.0127	0.0322
	60	0.0330	0.0142	0.0354
	80	0.0323	0.0170	0.0377
	100	0.0273	0.0181	0.0414
	200	0.0294	0.0189	0.0466

When *t* = 0.5, the original sequence is converted into a three-state Markov chain, and the type I error rates in the AR(1) model of different methods are presented in Table 4. In the AR(1) model, when *ρ*_1_ = −0.5, *ρ*_2_ = −0.5, the type I error rate of Permutation test still far exceeds the given significance level 0.05 even if the sample size is very small (*n* = 20), and TLTA cannot control the type I error rate even when the sample size is large. When *ρ*_1_ = 0, *ρ*_2_ = 0, the type I error rate of Permutation test is closer to the significance level than that of TLTA and STLTA, and the type I error rate of TLTA is far less than the significance level. When *ρ*_1_ > 0, *ρ*_2_ > 0, similar to the case of *t* = 0, the type I error rate of Permutation test also decreases with the increase of sample size *n*, and gradually deviates from the significance level. The type I error rate of TLTA is much smaller than the significance level, while that of STLTA shows an upward trend with the rise of the sample size *n* and gradually approaches the significance level. For different combinations of autocorrelation coefficients, the type I error rates of permutation test and TLTA decline with the increase of *ρ*, with a gradual deviation from the significance level, with TLTA in particular. Even though the autocorrelation is extremely weak, the type I error rate is far less than 0.05, even below 0.01. While STLTA performs well in controlling the type I error rate across all autocorrelation coefficient combinations. The performances of these three methods in ARMA(1,1) and ARMA(1,1)-TAR(1) models are shown in the Tables 5, 6. In these two models, the type I error rate of TLTA is always far less than the significance level. When *ρ*_1_ = −0.5, *ρ*_2_ = −0.5, the type I error rate of Permutation test is closer to the significance level than that of STLTA. But in other cases, the type I error rate of Permutation test is much smaller than the significance level, and it increasingly deviants from the significance level with the increase of sample size and autocorrelation, while the type I error rate of STLTA gradually approaches the significance level as the sample size increases.

**TABLE 4:** Type I error rate for different methods (the third to fifth columns) in the AR(1) model when*t* = 0.5. The first and second columns represent different combinations of autoregressive coefficients and sample sizes. The number of permutation tests is 1,000, the number of repeated simulations is 10,000, and the significance level is *α* = 0.05.

*ρ*_1_, *ρ*_2_	*n*	Permutation test	TLTA	STLTA
−0.5, −0.5	20	0.2236	0.0275	0.0400
	40	0.2155	0.0520	0.0134
	60	0.2210	0.0508	0.0119
	80	0.2158	0.0665	0.0166
	100	0.2159	0.0682	0.0178
	200	0.2213	0.0702	0.0226
0, 0	20	0.0737	0.0039	0.0263
	40	0.0628	0.0059	0.0188
	60	0.0594	0.0075	0.0220
	80	0.0572	0.0089	0.0247
	100	0.0552	0.0084	0.0246
	200	0.0580	0.0107	0.0325
0.3, 0.3	20	0.0379	0.0009	0.0276
	40	0.0296	0.0012	0.0216
	60	0.0296	0.0011	0.0277
	80	0.0229	0.0025	0.0304
	100	0.0270	0.0017	0.0324
	200	0.0241	0.0021	0.0398
0.3, 0.5	20	0.0243	0.0006	0.0229
	40	0.0174	0.0010	0.0246
	60	0.0170	0.0013	0.0263
	80	0.0184	0.0018	0.0337
	100	0.0184	0.0012	0.0334
	200	0.0152	0.0011	0.0355
0.5, 0.5	20	0.0196	0.0002	0.0175
	40	0.0149	0.0005	0.0221
	60	0.0102	0.0006	0.0282
	80	0.0105	0.0003	0.0311
	100	0.0124	0.0005	0.0350
	200	0.0104	0.0003	0.0430
0.5, 0.8	20	0.0099	0.0001	0.0159
	40	0.0052	0.0001	0.0194
	60	0.0036	0.0002	0.0286
	80	0.0032	0.0001	0.0303
	100	0.0033	0.0000	0.0325
	200	0.0017	0.0000	0.0377

**TABLE 5:** Type I error rate for different methods (the third to fifth columns) in the ARMA(1,1) model when*t* = 0.5. The first and second columns represent different combinations of autoregressive coefficients and sample sizes. The number of permutation tests is 1,000, the number of repeated simulations is 10,000, and the significance level is *α* = 0.05.

*ρ*_1_, *ρ*_2_	*n*	Permutation test	TLTA	STLTA
−0.5, −0.5	20	0.0767	0.0033	0.0269
	40	0.0609	0.0047	0.0166
	60	0.0595	0.0070	0.0212
	80	0.0566	0.0082	0.0229
	100	0.0542	0.0094	0.0284
	200	0.0552	0.0104	0.0343
0, 0	20	0.0300	0.0008	0.0251
	40	0.0211	0.0008	0.0354
	60	0.0187	0.0013	0.0429
	80	0.0201	0.0012	0.0442
	100	0.0185	0.0018	0.0456
	200	0.0190	0.0016	0.0533
0.3, 0.3	20	0.0137	0.0001	0.0239
	40	0.0112	0.0004	0.0395
	60	0.0115	0.0008	0.0424
	80	0.0083	0.0004	0.0453
	100	0.0100	0.0003	0.0489
	200	0.0073	0.0007	0.0579
0.3, 0.5	20	0.0109	0.0001	0.0208
	40	0.0073	0.0002	0.0306
	60	0.0044	0.0001	0.0431
	80	0.0044	0.0003	0.0456
	100	0.0048	0.0004	0.0473
	200	0.0037	0.0003	0.0565
0.5, 0.5	20	0.0076	0.0000	0.0206
	40	0.0050	0.0000	0.0360
	60	0.0052	0.0002	0.0406
	80	0.0041	0.0000	0.0442
	100	0.0041	0.0002	0.0511
	200	0.0028	0.0001	0.0509
0.5, 0.8	20	0.0020	0.0000	0.0148
	40	0.0010	0.0000	0.0249
	60	0.0011	0.0000	0.0288
	80	0.0008	0.0000	0.0333
	100	0.0007	0.0000	0.0333
	200	0.0003	0.0000	0.0470

**TABLE 6:** Type I error rate for different methods (the third to fifth columns) in the ARMA(1,1)-TAR(1) model when*t* = 0.5. The first and second columns represent different combinations of autoregressive coefficients and sample sizes. The number of permutation tests is 1,000, the number of repeated simulations is 10,000, and the significance level is *α* = 0.05.

*ρ*_1_, *ρ*_2_	*n*	Permutation test	TLTA	STLTA
−0.5, −0.5	20	0.0521	0.0013	0.0241
	40	0.0421	0.0034	0.0201
	60	0.0375	0.0040	0.0257
	80	0.0364	0.0049	0.0264
	100	0.0370	0.0049	0.0282
	200	0.0330	0.0049	0.0338
0, 0	20	0.0276	0.0005	0.0234
	40	0.0189	0.0009	0.0245
	60	0.0186	0.0009	0.0311
	80	0.0188	0.0009	0.0360
	100	0.0174	0.0011	0.0340
	200	0.0150	0.0016	0.0440
0.3, 0.3	20	0.0169	0.0003	0.0207
	40	0.0113	0.0005	0.0294
	60	0.0097	0.0007	0.0301
	80	0.0108	0.0006	0.0351
	100	0.0091	0.0007	0.0386
	200	0.0072	0.0004	0.0440
0.3, 0.5	20	0.0140	0.0000	0.0209
	40	0.0089	0.0005	0.0283
	60	0.0077	0.0000	0.0317
	80	0.0072	0.0006	0.0340
	100	0.0079	0.0003	0.0375
	200	0.0067	0.0004	0.0439
0.5, 0.5	20	0.0090	0.0001	0.0198
	40	0.0047	0.0001	0.0271
	60	0.0054	0.0000	0.0296
	80	0.0039	0.0002	0.0360
	100	0.0038	0.0002	0.0370
	200	0.0045	0.0000	0.0450
0.5, 0.8	20	0.0072	0.0000	0.0184
	40	0.0045	0.0001	0.0251
	60	0.0024	0.0001	0.0328
	80	0.0024	0.0001	0.0323
	100	0.0016	0.0000	0.0338
	200	0.0013	0.0000	0.0440

According to the analysis of the results, it can be figured out that STLTA is capable to control the type I error rate under different models, while the permutation test and TLTA are ineffective in this respect, which evidences that STLTA is more effective in utilizing the internal properties of time series than the other two methods, and that it can achieve a more accurate approximation of the local trend score *p* value.

### 3.2 Empirical Analysis

#### 3.2.1 Data set of Moving Pictures of Human Microbiome

**FIGURE 1:**
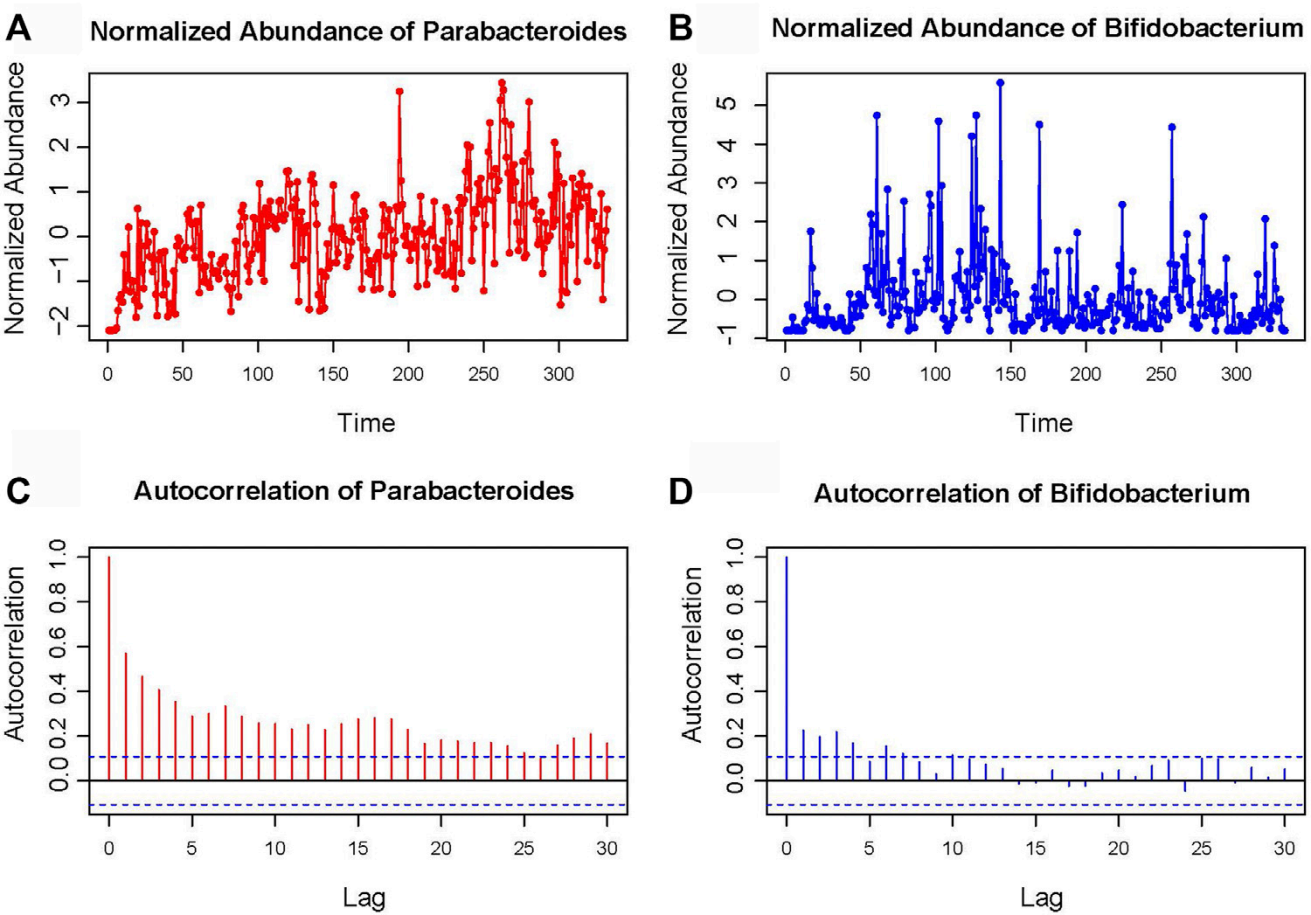
Standardized abundance map of *Parabacteroides* **(A)** and *Bifidobacterium* **(B)** in MPHM “M3” sample fecal data set. The autocorrelation graph **(C,D)** shows the autocorrelation coefficient of the time series at different delays.

The STLTA method is applied to the Moving Pictures of Human Microbiome (MPHM) data set, for comparison with the results as obtained from DDLSA, TLTA and Permutation test. The data set of MPHM was collected from two healthy subjects, one male (“M3”) and one female (“F4”). Both individuals were sampled daily at three body sites: gut (feces), mouth(tongue), and skin (left and right palms) (Caporaso et al. (2011)). The data set consists of 130, 135 and 133 daily samples from “F4”, and 332, 372 and 357 samples from “M3”. There are 335, 373 and 1,295 operational taxonomic units (OTUs) from feces, tongue and palm (both left and right) sites of “F4” and “M3”, where the taxonomic level is Genus. We selected 59 “core” OTUs that were observed in at least 60% samples from the feces of “M3” and analyzed their relationships. Then, metagenomic analysis is conducted to obtain a time series of OTU abundance. As shown in Figure 1, there are two OTUs chosen to display their time series graphs and autocorrelation graphs. It can be found that the abundance sequence of Parabacteroides shows more significant autocorrelation compared to Bifidobacterium, and that their Box-Ljung test *p* values are all very close to 0, indicating that their autocorrelation relationship is of much significance.

The significance level is set to 0.05 and 0.01, based on which a comparison is drawn in the significant relationship between the OTUs found by permutation test, TLTA, STLTA and DDLSA with the time delay of *D* = 3. The results are presented in Table 7. When *t* = 0.5 and the significance level *p* = 0.05, *Q* = 0.05, in all 1711 pairs of OTU relationships in the “M3” feces sample, it was found that 589, 165, 739 and 685 pairs of significant relationships by Permutation test, TLTA, STLTA and DDLSA respectively, which were 34.4, 9.6, 43.2 and 40% of the total. STLTA found the most significant relationship, followed by DDLSA, and TLTA the least. This is very similar to the simulation results obtained earlier: when *t* = 0.5 and the sample time point is 300, if the samples have autocorrelation relationship, the simulation results show that the type I error rates of Permutation test and TLTA are far less than the given significance level, while the type I error rate of STLTA is close to the given significance level. Therefore when there is correlation between autocorrelation samples, it is possible that permutation test and TLTA fail to identify many significant relationships that actually exist, but STLTA can do this. Although the permutation test can also find many significant relationships, most of them are between samples without autocorrelation. In addition, the numbers of significant correlations between OTUs found by STLTA and DDLSA are approximate, shown that STLSA can discover most significant relationships found by DDLSA.

**TABLE 7:** The numbers of significant correlations between OTUs found by permutation tests, TLTA, STLTA and DDLSA for different data sets and significance levels.

	—	t = 0.5		t = 0	
Dataset	—	MPHM	PML	MPHM	PML
# of factors	—	59	75	59	75
*p* ≤ 0.05 *q* ≤ 0.05	Permutation	589	87	727	29
—	TLTA	165	75	532	13
—	STLTA	739	50	667	13
—	DDLSA	685	371	685	371
*p* ≤ 0.01 *q* ≤ 0.01	Permutation	489	84	549	29
—	TLTA	86	74	436	11
—	STLTA	621	16	514	4
—	DDLSA	549	227	549	227

**FIGURE 2:**
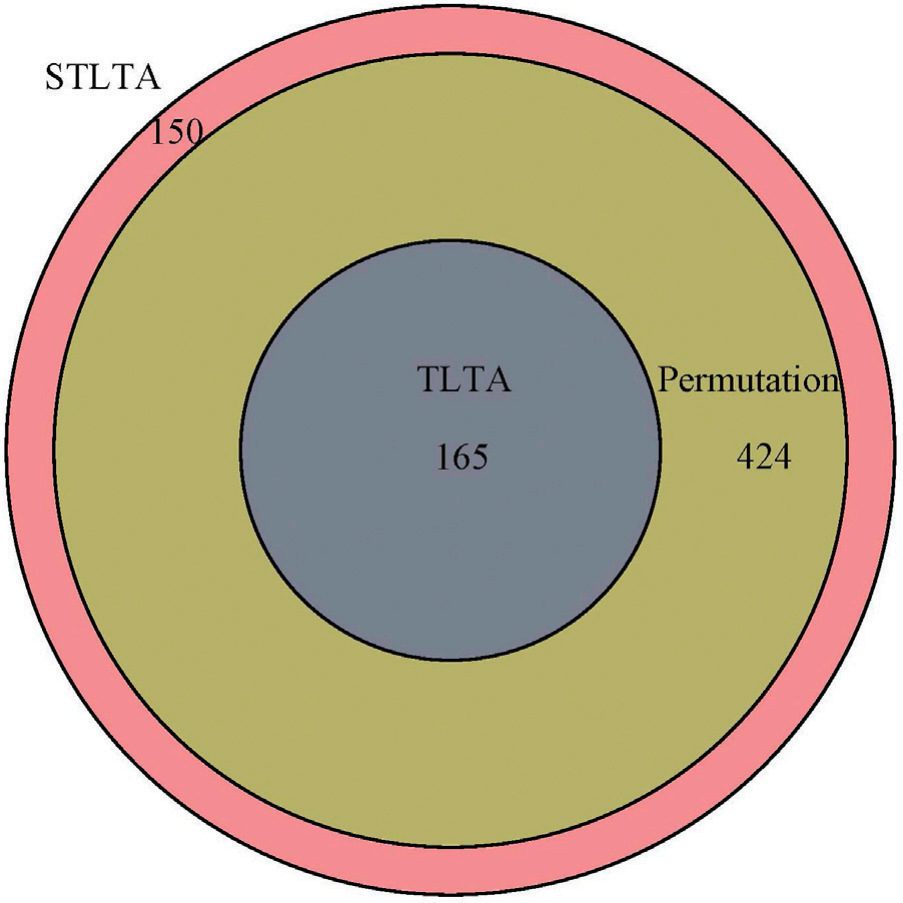
The Venn diagram of the significant relationships found in permutation test, TLTA and STLTA for the MPHM “M3” fecal data set. Green, blue, and red indicate the number of significant relationships found by permutation test, TLTA, and STLTA, respectively.

Venn diagram (Figure 2) shows the relationship among the results obtained using different methods in the “M3” stool sample. All of the significant relationships identified by TLTA are discovered by permutation test, and all of the significant relationships identified by permutation test are discovered by STLTA. For more stringent standards *p* = 0.01 and *Q* = 0.01 as well as different thresholds, the results are listed in Table 7. By comparing the results of *t* = 0 and *t* = 0.5, it can be found out that the permutation test and TLTA can identify more significant relationships at *t* = 0 then at *t* = 0.5, especially for TLTA. However, STLTA is just the opposite, with the significant relationship found at *t* = 0 less then at *t* = 0.5.

#### 3.2.2 Data set of Plymouth Marine Laboratory

The STLTA method is applied to the Plymouth Marine Laboratory (PML) data set, for comparison with the results as obtained from DDLSA, TLTA and Permutation test. The PML data set is one of the longest microbial time series consisting of monthly samples taken over 6 years at a temperate marine coastal site off Plymouth, United Kingdom (Gilbert et al. (2012)). These samples were sequenced using high-resolution 16S rRNA tag NGS sequencing. A total of 155 bacterial OTUs were identified with the taxonomic level of Order. Among them, we chose 62 abundant OTUs that were present in at least 50% of the time points, and 13 environment factors to analyze their association network. We filled the missing values in the environment data using linear interpolation.

**FIGURE 3:**
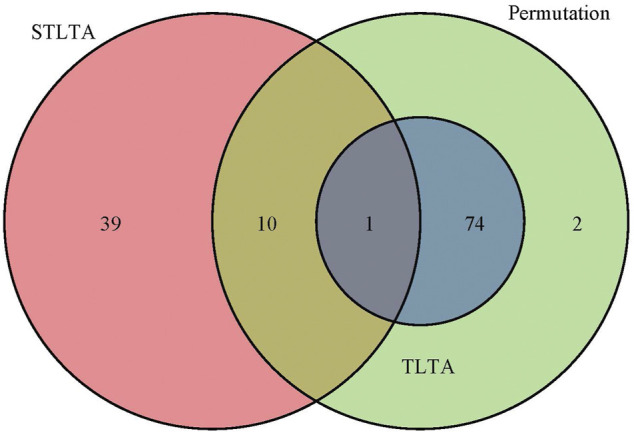
The Venn diagram of the significant relationships found in permutation test, TLTA and STLTA for the PML data set. Green, blue, and red indicate the number of significant relationships found by permutation test, TLTA, and STLTA, respectively.

Given time delay *D* = 3 and significance level *p* = 0.05, *Q* = 0.05, when *t* = 0.5 among all the relationships between OTUs and between OTU and environmental factors, permutation test, TLTA, STLTA and DDLSA identified 87, 75, 50 and 371 pairs of significant relationships, as shown in Table 7. Venn diagram (Figure 3) reveals the relationships among the results as obtained using different methods in the PML samples. All of the significant relationships identified by TLTA are discovered by permutation tests. Among all these significant relationships, however, only 11 pairs of relationships are found out by both permutation test and STLTA. This is because there are only 33 (∼44*%*) factors showing autocorrelation, with more than half of the factors bearing no autocorrelation. Therefore, permutation test can be conducted to find out about the significant relationships between many time series without autocorrelation. However, there are as few as 72 sample time points, since STLTA is conservative to some extent when there are a small number of time points. Among the significant relationships discovered by the permutation test, there are 76 pairs not identified by STLTA. In addition, it is suspected that 39 pairs of significant relationships which are found out by STLTA but fail to be detected by permutation test are between autocorrelation sequences, and these relationships can be discovered by neither permutation test nor TLTA. For more stringent standards *p* = 0.01 and *Q* = 0.01 as well as different thresholds, the results are shown in Table 7. It can be found out from the table that when *t* = 0, the number of significant relationships identified by all methods is smaller than that of relationships discovered when *t* = 0.5. As the PML data set has only 72 time points, there is a massive information loss in STLTA. Thus, the number of significant correlations between OTUs found by STLTA is far from that by DDLSA.

## 4 Conclusion

In this paper, a theoretical evaluation method was proposed for the statistical significance of local trend scores, STLTA. First of all, the original sequence was discretized into a changing trend sequence and the local trend score was calculated. Then, according to the spectral decomposition theory of the matrix, the variance of the trend sequence was estimated for different state spaces. Finally, in combination with the limit theory of Markov chain local similarity analysis, the limit distribution of the local trend score was obtained, and the approximate *p* value of the local trend score was calculated. By means of simulation, it was discovered in a given stationary time series model that the type I error rate of STLTA can be made significantly closer to the given significance level, with the type I error rates of permutation test and TLTA increasingly deviant from the given significance level over time, especially when *t* = 0.5. It is suggested that STLTA method is more effective than permutation test and TLTA method. Then, these three methods were applied to the MPHM and PML data sets. In the relatively long data set MPHM “M3” fecal data set, STLTA detected the most significant relationships, and all of the significant relationships discovered by permutation tests and TLTA were identified by STLTA. In the PML data set with relatively short time points, STLTA discovered some relationships that cannot be found out by permutation tests and TLTA, with these relationships resulting from the autocorrelation of the sequence.

Compared with local similarity analysis, however, local trend analysis converts a continuous original time series into a discrete trend series, which may cause the loss of some information from the original series, thus limiting the practical application of local trend analysis. Nonetheless, the discretization of the original sequence may lead to the transformation of some non-stationary time series into a stationary Markov sequence, which is a major advantage of local trend analysis. In addition, the DDLSA based on non-parametric kernel estimation and the MBBLSA based on moving block bootstrap can be applied to the statistical significance analysis as part of local trend analysis, which provides another direction of further research.

## Data Availability

Publicly available datasets were analyzed in this study. The "MPHM" datasets used during the current study are publicly available in the supplementary of Gilbert et al. (2012), whose link is https://genomebiology.biomedcentral.com/articles/10.1186/gb-2011-12-5-r50#additional-information. The "PML" data can be found here: https://vamps2.mbl.edu/.
