# Supplementary material for: Efficient Approximation of Statistical Significance in Local Trend Analysis of Dependent Time Series
Source: Front Genet. 2022 Apr 26;13:729011. doi: 10.3389/fgene.2022.729011 (PMC9086404; doi:10.3389/fgene.2022.729011)
Supplement: Supplementary file 1 [file DataSheet1.PDF]

Supplement

Sup1

It can be obtained by calculation, $det(\lambda \boldsymbol{I} - \boldsymbol{T}_X) = (\lambda - 1)(\lambda - (2a_X - 1))$, so the eigenvalues of $\boldsymbol{T}_X$ are $\lambda_1 = 1, \lambda_2 = 2a_X - 1$. The right eigenvector corresponding to λ_1 is $\boldsymbol{r}_1 = \left(\frac{1}{\sqrt{2}}, \frac{1}{\sqrt{2}}\right)^T$, the left eigenvector is $\boldsymbol{\ell}_1^T = \left(\frac{1}{\sqrt{2}}, \frac{1}{\sqrt{2}}\right)$. The right eigenvector corresponding to λ_2 is $\boldsymbol{r}_2 = \left(\frac{1}{\sqrt{2}}, -\frac{1}{\sqrt{2}}\right)^T$, the left eigenvector is $\boldsymbol{\ell}_2^T = \left(\frac{1}{\sqrt{2}}, -\frac{1}{\sqrt{2}}\right)$. Since $\boldsymbol{T}_X$ is a symmetric matrix, by the definition of simple matrix, $\boldsymbol{T}_X$ is a simple matrix. So it can be obtained from the Spectral decomposition theorem that

$$\begin{aligned}
\boldsymbol{T}_X^k &= \lambda_1^k \boldsymbol{r}_1 \boldsymbol{\ell}_1^T + \lambda_2^k \boldsymbol{r}_2 \boldsymbol{\ell}_2^T \\
&= \begin{pmatrix} \frac{1}{\sqrt{2}} \\ \frac{1}{\sqrt{2}} \end{pmatrix} \begin{pmatrix} \frac{1}{\sqrt{2}} & \frac{1}{\sqrt{2}} \end{pmatrix} + (2a_X - 1)^k \begin{pmatrix} \frac{1}{\sqrt{2}} \\ -\frac{1}{\sqrt{2}} \end{pmatrix} \begin{pmatrix} \frac{1}{\sqrt{2}} & -\frac{1}{\sqrt{2}} \end{pmatrix} \\
&= \begin{pmatrix} \frac{1}{2} + \frac{1}{2}(2a_X - 1)^k & \frac{1}{2} - \frac{1}{2}(2a_X - 1)^k \\ \frac{1}{2} - \frac{1}{2}(2a_X - 1)^k & \frac{1}{2} + \frac{1}{2}(2a_X - 1)^k \end{pmatrix} \\
&= \frac{1}{2} \begin{pmatrix} 1 + (2a_X - 1)^k & 1 - (2a_X - 1)^k \\ 1 - (2a_X - 1)^k & 1 + (2a_X - 1)^k \end{pmatrix}.
\end{aligned} \tag{1}$$

When $k \geq 1$,

$$\begin{aligned}
P(d_1^X d_{k+1}^X = 1) = P(d_{k+1}^X = 1 | d_1^X = 1) P(d_1^X = 1) + \\
P(d_{k+1}^X = -1 | d_1^X = -1) P(d_1^X = -1) \\
= \frac{1}{2}\left(1 + (2a_X - 1)^k\right) \times \frac{1}{2} + \frac{1}{2}\left(1 + (2a_X - 1)^k\right) \times \frac{1}{2} \\
= \frac{1}{2}\left(1 + (2a_X - 1)^k\right).
\end{aligned} \tag{2}$$

$$\begin{aligned}
P(d_1^X d_{k+1}^X = -1) &= P(d_{k+1}^X = -1 | d_1^X = 1) P(d_1^X = 1) + P(d_{k+1}^X = 1 | d_1^X = -1) P(d_1^X = -1) \\
&= \frac{1}{2}\left(1 - (2a_X - 1)^k\right) \times \frac{1}{2} + \frac{1}{2}\left(1 - (2a_X - 1)^k\right) \times \frac{1}{2} \\
&= \frac{1}{2}\left(1 - (2a_X - 1)^k\right).
\end{aligned} \tag{3}$$

So,

$$\begin{aligned}
\mathbb{E}(d_1^X d_{k+1}^X) &= \frac{1}{2}(1 + (2a_X - 1)^k) - \frac{1}{2}(1 - (2a_X - 1)^k) \\
&= (2a_X - 1)^k.
\end{aligned} \tag{4}$$

In the same way, the stationary distribution of d_i^Y is $P(d_i^Y = 1) = P(d_i^Y = -1) = 1/2, \mathbb{E}((d_1^Y)^2) = 1$, $\mathbb{E}(d_1^Y d_{k+1}^Y) = (2a_Y - 1)^k$.

Sup2 It can be obtained by calculation, $det(\lambda \boldsymbol{I} - \boldsymbol{T}_X) = (\lambda - 1)(\lambda - (b_X + c_X - 2d_X))(\lambda - (b_X - c_X))$, so the eigenvalues of $\boldsymbol{T}_X$ are $\lambda_1 = 1, \lambda_2 = b_X + c_X - 2d_X, \lambda_3 = b_X - c_X$. The right eigenvector corresponding to λ_1 is $\boldsymbol{r}_1 = (1, 1, 1)^T$, the left eigenvector is $\boldsymbol{\ell}_1^T = \left(\frac{d_X}{1 - b_X - c_X + 2d_X}, \frac{1 - b_X - c_X}{1 - b_X - c_X + 2d_X}, \frac{d_X}{1 - b_X - c_X + 2d_X}\right)$. The right eigenvector corresponding to λ_2 is $\boldsymbol{r}_2 = \left(1, -\frac{2d_X}{1 - b_X - c_X}, 1\right)^T$, the left eigenvector is $\boldsymbol{\ell}_2^T = \left(\frac{1 - b_X - c_X}{2(1 - b_X - c_X + 2d_X)}, -\frac{1 - b_X - c_X}{1 - b_X - c_X + 2d_X}, \frac{1 - b_X - c_X}{2(1 - b_X - c_X + 2d_X)}\right)$. The right eigenvector corresponding to λ_3 is $\boldsymbol{r}_3 = (1, 0, -1)^T$, the left eigenvector is $\boldsymbol{\ell}_3^T = (\frac{1}{2}, 0, -\frac{1}{2})$. Since the elements of the transition probability matrix are all non-negative values, $0 < 1 - b_X - c_X < 1, 0 < 1 - 2d_X < 1$, thus $0 < 1 - b_X - c_X + 2d_X < 2$.

It can be obtained by simple calculations, the right (left) eigenvectors of $\boldsymbol{T}_X$ are linearly independent, and the algebraic multiplicity of each eigenvalue is the same as the geometric multiplicity, so $\boldsymbol{T}_X$ is a simple matrix. It can be obtained from the Spectral decomposition theorem that

$$
\begin{aligned}
\boldsymbol{T}_X^k &= \lambda_1^k \boldsymbol{r}_1 \boldsymbol{\ell}_1^T + \lambda_2^k \boldsymbol{r}_2 \boldsymbol{\ell}_2^T + \lambda_3^k \boldsymbol{r}_3 \boldsymbol{\ell}_3^T \\
&= \begin{pmatrix} 1 \\ 1 \\ 1 \end{pmatrix} \begin{pmatrix} \frac{d_X}{1-b_X-c_X+2d_X} & \frac{1-b_X-c_X}{1-b_X-c_X+2d_X} & \frac{d_X}{1-b_X-c_X+2d_X} \end{pmatrix} + (b_X + c_X - 2d_X)^k \begin{pmatrix} 1 \\ -\frac{2d_X}{1-b_X-c_X} \\ 1 \end{pmatrix} \times \\
&\begin{pmatrix} \frac{1-b_X-c_X}{2(1-b_X-c_X+2d_X)} & -\frac{1-b_X-c_X}{1-b_X-c_X+2d_X} & \frac{1-b_X-c_X}{2(1-b_X-c_X+2d_X)} \end{pmatrix} + (b_X - c_X)^k \begin{pmatrix} 1 \\ 0 \\ -1 \end{pmatrix} \begin{pmatrix} \frac{1}{2} & 0 & -\frac{1}{2} \end{pmatrix} \\
&= \begin{pmatrix} T_{-1,-1}^{X,k} & T_{-1,0}^{X,k} & T_{-1,1}^{X,k} \\ T_{0,-1}^{X,k} & T_{0,0}^{X,k} & T_{0,1}^{X,k} \\ T_{1,-1}^{X,k} & T_{1,0}^{X,k} & T_{1,1}^{X,k} \end{pmatrix}. \qquad (5)
\end{aligned}
$$

Where

$$
\begin{aligned}
T_{-1,-1}^{X,k} &= \frac{d_X}{1-b_X-c_X+2d_X} + \frac{(1-b_X-c_X)(b_X+c_X-2d_X)^k}{2(1-b_X-c_X+2d_X)} + \frac{1}{2}(b_X-c_X)^k, \\
T_{-1,0}^{X,k} &= \frac{1-b_X-c_X}{1-b_X-c_X+2d_X} - \frac{(1-b_X-c_X)(b_X+c_X-2d_X)^k}{1-b_X-c_X+2d_X}, \\
T_{-1,1}^{X,k} &= \frac{d_X}{1-b_X-c_X+2d_X} + \frac{(1-b_X-c_X)(b_X+c_X-2d_X)^k}{2(1-b_X-c_X+2d_X)} - \frac{1}{2}(b_X-c_X)^k, \\
T_{0,-1}^{X,k} &= \frac{d_X}{1-b_X-c_X+2d_X} - \frac{d_X(b_X+c_X-2d_X)^k}{1-b_X-c_X+2d_X}, \\
T_{0,0}^{X,k} &= \frac{1-b_X-c_X}{1-b_X-c_X+2d_X} + \frac{2d_X(b_X+c_X-2d_X)^k}{1-b_X-c_X+2d_X}, \\
T_{0,1}^{X,k} &= \frac{d_X}{1-b_X-c_X+2d_X} - \frac{d_X(b_X+c_X-2d_X)^k}{1-b_X-c_X+2d_X}, \\
T_{1,-1}^{X,k} &= \frac{d_X}{1-b_X-c_X+2d_X} + \frac{(1-b_X-c_X)(b_X+c_X-2d_X)^k}{2(1-b_X-c_X+2d_X)} - \frac{1}{2}(b_X-c_X)^k, \\
T_{1,0}^{X,k} &= \frac{1-b_X-c_X}{1-b_X-c_X+2d_X} - \frac{(1-b_X-c_X)(b_X+c_X-2d_X)^k}{1-b_X-c_X+2d_X}, \\
T_{1,1}^{X,k} &= \frac{d_X}{1-b_X-c_X+2d_X} + \frac{(1-b_X-c_X)(b_X+c_X-2d_X)^k}{2(1-b_X-c_X+2d_X)} + \frac{1}{2}(b_X-c_X)^k.
\end{aligned} \qquad (6)
$$

Let $k \to \infty$, the stationary distribution of d_i^X can be obtained that

$$
\begin{aligned}
\varphi_X &= \left(\frac{d_X}{1-b_X-c_X+2d_X}, \frac{1-b_X-c_X}{1-b_X-c_X+2d_X}, \frac{d_X}{1-b_X-c_X+2d_X} \right) \\
&= (\varphi_{-1}^X, \varphi_0^X, \varphi_1^X).
\end{aligned} \qquad (7)
$$

So,

$$
\begin{aligned}
P(d_1^X d_{k+1}^X = 1) &= P(d_{k+1}^X = 1 | d_1^X = 1) P(d_1^X = 1) \\
&\quad + P(d_{k+1}^X = -1 | d_1^X = -1) P(d_1^X = -1) \\
&= \varphi_1^X T_{1,1}^{X,k} + \varphi_{-1}^X T_{-1,-1}^{X,k}.
\end{aligned} \qquad (8)
$$

The same can be obtained,

$$
\begin{aligned}
P(d_1^X d_{k+1}^X = -1) &= P(d_{k+1}^X = -1 | d_1^X = 1) P(d_1^X = 1) \\
&\quad + P(d_{k+1}^X = 1 | d_1^X = -1) P(d_1^X = -1) \\
&= \varphi_1^X T_{1,-1}^{X,k} + \varphi_{-1}^X T_{-1,1}^{X,k}.
\end{aligned} \qquad (9)
$$

Thus,

$$
\mathbb{E}(d_1^X d_{k+1}^X) = \varphi_1^X T_{1,1}^{X,k} + \varphi_{-1}^X T_{-1,-1}^{X,k} - \varphi_1^X T_{1,-1}^{X,k} - \varphi_{-1}^X T_{-1,1}^{X,k}. \qquad (10)
$$

In addition, it can be obtained by the stable distribution that

$$
\mathbb{E}((d_1^X)^2) = \varphi_{-1}^X + \varphi_1^X. \qquad (11)
$$

Similarly,

$$
\begin{aligned}
\varphi_Y &= (\varphi_{-1}^Y, \varphi_0^Y, \varphi_1^Y) \\
&= \left(\frac{d_Y}{1 - b_Y - c_Y + 2d_Y}, \frac{1 - b_Y - c_Y}{1 - b_Y - c_Y + 2d_Y}, \frac{d_Y}{1 - b_Y - c_Y + 2d_Y} \right). \\
\mathbb{E}((d_1^Y)^2) &= \varphi_{-1}^Y + \varphi_1^Y. \\
\mathbb{E}(d_1^Y d_{k+1}^Y) &= \varphi_1^Y T_{1,1}^{Y,k} + \varphi_{-1}^Y T_{-1,-1}^{Y,k} - \varphi_1^Y T_{1,-1}^{Y,k} - \varphi_{-1}^Y T_{-1,1}^{Y,k}.
\end{aligned}
$$
